# Effective Connectivity of Hippocampal Neural Network and Its Alteration in Mg^2+^-Free Epilepsy Model

**DOI:** 10.1371/journal.pone.0092961

**Published:** 2014-03-21

**Authors:** Xin-Wei Gong, Jing-Bo Li, Qin-Chi Lu, Pei-Ji Liang, Pu-Ming Zhang

**Affiliations:** 1 School of Biomedical Engineering, Shanghai Jiao Tong University, Shanghai, China; 2 Department of Neurology, Ren Ji Hospital, Shanghai Jiao Tong University, Shanghai, China; McGill University, Canada

## Abstract

Understanding the connectivity of the brain neural network and its evolution in epileptiform discharges is meaningful in the epilepsy researches and treatments. In the present study, epileptiform discharges were induced in rat hippocampal slices perfused with Mg^2+^-free artificial cerebrospinal fluid. The effective connectivity of the hippocampal neural network was studied by comparing the normal and epileptiform discharges recorded by a microelectrode array. The neural network connectivity was constructed by using partial directed coherence and analyzed by graph theory. The transition of the hippocampal network topology from control to epileptiform discharges was demonstrated. Firstly, differences existed in both the averaged in- and out-degree between nodes in the pyramidal cell layer and the granule cell layer, which indicated an information flow from the pyramidal cell layer to the granule cell layer during epileptiform discharges, whereas no consistent information flow was observed in control. Secondly, the neural network showed different small-worldness in the early, middle and late stages of the epileptiform discharges, whereas the control network did not show the small-world property. Thirdly, the network connectivity began to change earlier than the appearance of epileptiform discharges and lasted several seconds after the epileptiform discharges disappeared. These results revealed the important network bases underlying the transition from normal to epileptiform discharges in hippocampal slices. Additionally, this work indicated that the network analysis might provide a useful tool to evaluate the neural network and help to improve the prediction of seizures.

## Introduction

Epilepsy is a neurological disorder of the brain function characterized by recurrent unprovoked discharges in large aggregates of neurons. Because seizures involve complex interactions across several regions of the brain, investigating the regional interactions during the evolution of seizures may help to understand the pathophysiological changes of the neural network, and provide meaningful guidance for the epilepsy therapy [1].

Neural networks are mainly described by anatomical, functional and effective connectivity [2], [3]. The anatomical connectivity represents physical connections, i.e. chemical synapses, electrical synapses, etc. The functional connectivity demonstrates the symmetrical statistical dependence between the activities of pairs of nodes. The effective connectivity reflects the directed or causal influence of one node on another. The analyses on neural network connectivity have been carried out extensively in human brain based on various measurements, such as electroencephalogram (EEG), magnetoencephalogram (MEG), functional magnetic resonance image (fMRI), diffusion tensor image (DTI) and so on [4]–[6], providing valuable knowledge on the brain functions, disease diagnosis, etc. For the epilepsy researches, neural network characteristics, such as out-degree [7], betweenness centrality [8], small-world property [9]–[11], have been used to localize the seizure-onset zone [7], [8] and inspect the alteration of network connectivity patterns in the interictal state [11], [12]. These researches were performed on large-scale brain networks with relatively low spatial resolution, which might result in low precision of the spatial properties of the networks. The investigation of the network topology on smaller, localized brain regions using the technique with higher spatial resolution might provide more detailed information about the network. The microelectrode array (MEA) is an ideal equipment to record signals with high spatial and temporal resolution [13], and has been employed to investigate the initiation, propagation, and spatiotemporal patterns of the epileptiform discharges in rat hippocampal slices [14]–[16], as well as the effects of anti-epilepsy drugs [17], [18].

Hippocampus plays an important role in the temporal lobe epilepsy (TLE). The hippocampal neural network is a complex network containing a large amount of neurons distributed in several subfields and layers, with these subareas being interconnected in a complex way. For TLE patients, the abnormal electrical activities were often detected in the hippocampus [19]. Moreover, neuroanatomical researches found that TLE was often related to the mossy fiber sprouting, loss of neurons in CA1 subfield, and some other changes [20]. Highly interconnected hubs were found existing in the dentate gyrus (DG) of epileptic rats and might contribute to the initiation and propagation of the epileptiform discharges in the epileptogenic networks [21]. Researches on the hippocampal slices of developing rats revealed that the network followed a scale-free topology and the highly connected hubs were a subpopulation of interneurons [22]. The hippocampal network might change its effective connections among the local circuits during the transition from normal to epileptiform discharges. However, the quantitative analysis focused on the alteration of the network connectivity during the transition process is still limited.

In the present study, epileptiform discharges in rat hippocampal slices were induced by Mg^2+^-free artificial cerebrospinal fluid (ACSF) and recorded by the MEA. The networks during control and epileptiform discharges were constructed from the association matrix formed by partial directed coherence (PDC) and the network characteristics were analyzed by graph theory. The results increased the knowledge on the functional organization of the hippocampal neural network and might help to better understand the network transition from normal to epileptiform discharges in the hippocampus.

## Materials and Methods

### Ethics statement

The animal experiments were approved by the Ethics Committee, School of Biomedical Engineering, Shanghai Jiao Tong University. All efforts were made to minimize the number of animals used and their suffering.

### Hippocampal slice preparation

Eight male Sprague-Dawley (SD) rats at postnatal day 23–25 were used in our experiments. After decapitation, the brain was rapidly dissected and placed in oxygenated (95% O_2_/5% CO_2_) ice-cold ACSF containing (in mM): NaCl 124.0, NaHCO_3_ 25.0, KCl 3.5, CaCl_2_ 2.5, MgCl_2_•6H_2_O 1.3, NaH_2_PO_4_ 1.2, and glucose 10.0. pH of the solution was adjusted to 7.4 with 1.0 M HCl. The osmolarity was approximately 300 mOsm/L. Transverse hippocampal slices (400 μm) were prepared using a mechanical vibratome (Series 1000, Tissue Sectioning System, Vibratome, Natural Genetic Ltd., USA) and incubated in oxygenated ACSF at 25–27°C for at least 2 hours before recording. The Mg^2+^-free ACSF was prepared to induce the epileptiform discharges by omitting MgCl_2_•6H_2_O from the ACSF without substitution.

### Electrophysiological recordings

**Figure 1:**
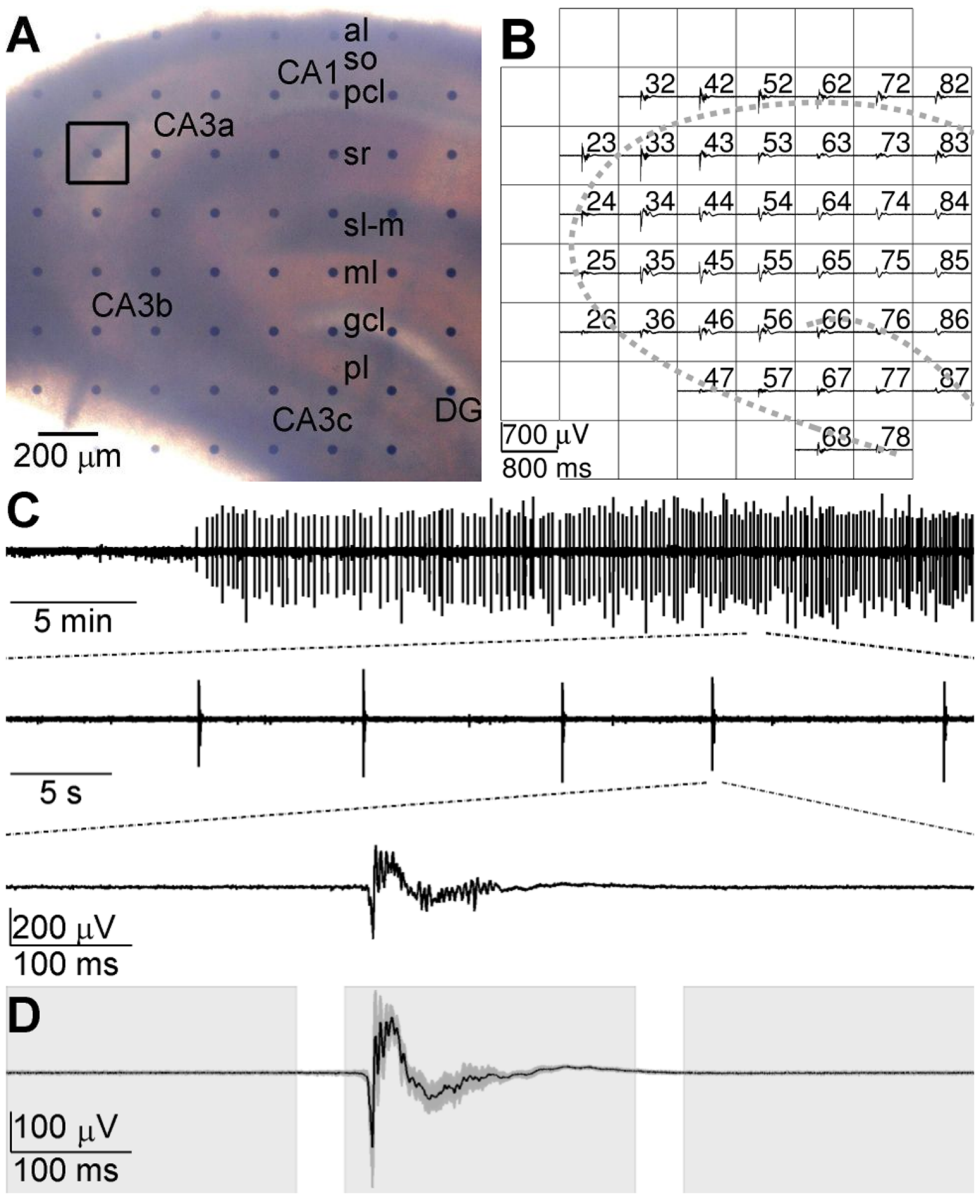
Epileptiform discharges recorded by MEA. A: An example of hippocampal slice mounted on MEA. CA1, CA3a, CA3b and CA3c, fields of the hippocampus; DG, dentate gyrus; al, alveus; so, stratum oriens; pcl, pyramidal cell layer; sr, stratum radiatum; sl-m, stratum lacunosum moleculare; ml, molecular layer; gcl, granule cell layer; pl, polymorphic layer. B: Simultaneous extracellular recording of the hippocampal slice at about 30 min after the start of Mg^2+^-free ACSF perfusion. The channel number of each electrode is labeled at its top right corner. Several channels were closed (blank grids) because of a low signal-to-noise ratio. The two gray dashed curves illustrate the positions of pyramidal cell layer and granule cell layer. C: Epileptiform discharges displayed in different time scales. The epileptiform discharges were recorded by electrode #23, which is indicated by a black square in (A). D: Ensemble mean (black line) and standard deviation (dark gray shade) of 50 epileptiform discharges. Three blocks of light shades indicate the early, middle and late stages of epileptiform discharges.

The neural electrical activities were acquired by the multi-channel recording system (MEA60, Multi Channel Systems GmbH, Germany). The MEA consists of 60 planar electrodes arranged in an 8×8 array with diameter of 30 μm (leaving the 4 corners void). The horizontal and vertical tip-to-tip distance between neighboring electrodes is 200 μm as shown in Fig. 1A. The hippocampal slice was placed in the electrode area (1.4 mm×1.4 mm) of the MEA and covered by a nylon mesh to make a better contact with the electrodes. The slice was perfused with oxygenated ACSF at a rate of 2 ml/min with a peristaltic pump. The temperature of perfusate at MEA chamber was maintained at 25–27°C with a temperature control unit (TC01, Multi Channel Systems GmbH, Germany). After the slice was perfused for at least 10 min, the normal ACSF was changed to Mg^2+^-free ACSF to induce epileptiform discharges. The repetitive synchronized epileptiform discharges were observed in most hippocampal regions after 10–30 min of the Mg^2+^-free ACSF perfusion. After 1200× amplification (bandwidth 1 Hz to 3 kHz) by a 60-channel amplifier (MEA1060, Multi Channel Systems GmbH, Germany), the signals were sampled at a rate of 20 kHz and stored for offline analysis.

### Data Preprocessing

In our experiments, the epileptiform discharges induced by Mg^2+^-free ACSF were observed in most recording channels in hippocampal slices (Fig. 1B). A threshold of 10 times the standard deviation of the background signal was set to detect the epileptiform discharges in one channel in the pyramidal cell layer which had a high signal-to-noise ratio. Once the signal of the selected channel exceeded the threshold, 800-ms data segments (300 ms pre- and 500 ms post- the detected time point) in all of the channels at the same time episode were taken as epileptiform discharges. The continuous 800-ms segments of signals prior to Mg^2+^-free ACSF perfusion which did not contain apparent population spikes were used to study the hippocampal networks in control. After the control and epileptiform discharges were detected, a notch filtering was performed to filter out the 50 Hz power noise. Then the temporal mean was subtracted and the standard deviation was divided from each data segment [23]. The difference of the functional organization of the neural network during control and epileptiform discharges was investigated.

### Partial Directed Coherence

PDC is a method to determine the causal interaction between multivariate time series based on the multivariate autoregressive (MVAR) model, which was first introduced by Sameshima and Baccala in 1999 [24]. PDC was used in our present study to generate the association matrix of the hippocampal neural network.

MVAR model analysis requires that the time series are stationary. For our signals, which were not stationary, the strategy that Ding et al. used to process the event-related potentials was adopted [23]. Two sources of nonstationarity were removed by two steps. Firstly, the nonstationarity embodied in the mean and standard deviation was removed by subtracting the ensemble mean and being divided by the ensemble standard deviation. Here, each epileptiform discharge was considered as a realization of one stochastic process. When the epileptiform discharges steadily appeared (usually 30 min after the Mg^2+^-free ACSF perfusion began), we continuously sampled 50 epileptiform discharges from each recording channel. The ensemble mean and the ensemble standard deviation of the signals were calculated across all the 50 epileptiform discharges at each time point for each channel. Secondly, the correlation nonstationarity was removed by using short time windows in which the underlying stochastic processes were considered to be locally stationary. Here, highly overlapped short time windows (window size: 50 ms, sliding step: 10 ms) were used. Meanwhile, the same processing procedure was applied to data segments collected in the control condition. The preprocessed signals were used to construct the MVAR model as follows.

Let 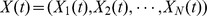 be a set of the preprocessed signals of neural activities. Here 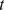 refers to the time and 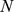 is the number of recording channels. The signals in all recording channels were used except for a few ones with a low signal-to-noise ratio or recorded by the electrodes that were not covered by the slice.

The MVAR model takes the form
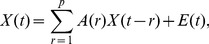
(1)where 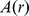 are 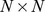 coefficient matrices to be estimated; 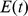 is a vector of multivariate zero-mean white noise; 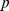 is the model order which was determined by Akaike information criterion (AIC) [25] defined as
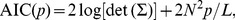
(2)where det(Σ) denotes the determinant of the covariance matrix of 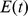 and 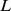 is the number of the data points applied in the estimation. To estimate 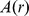, Equation (1) is multiplied from the right by 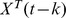, where 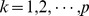. Taking expectation, we obtain the Yule-Walker equations
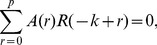
(3)where 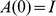, with 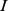 being the 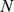-dimensional identity matrix; 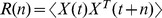 is 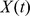
's covariance matrix of lag *n*. For multiple realizations, we compute 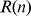 for each realization and average across all the realizations to obtain the final estimate of the covariance matrix. Then 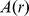 is obtained using the Levison-Wiggins-Robinson (LWR) algorithm [26].

After the Fourier transformation of the MVAR coefficients
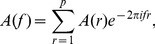
(4)the frequency-domain representation of the MVAR model was obtained. Define the matrix 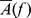 as
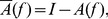
(5)


Then PDC value at frequency *f* from variable 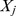 to 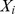 is defined as
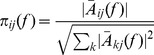
(6)


The 3-demesional PDC values depict causal relationships among all channels at different frequencies. Statistical significance test was performed in order to remove the spurious interactions between channels [27]. A Fourier-transformed surrogate data method [28] was applied to each channel. Then the PDC method was applied on the surrogate data. After repeating that procedure 1000 times, a distribution of PDC values of surrogate data was obtained that corresponded to the null hypothesis of no causal interactions. A significance level was set (*p* = 0.05) for the statistical test and the links in the network with the connecting strengths below the threshold were discarded.

After the spurious links were eliminated, the maximal PDC values along the frequency dimension in a low frequency band (1–300 Hz) were selected and transposed to obtain the association matrix
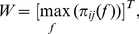
(7)where the superscript *T* denotes matrix transposition. Each element 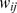 in the 2-dimensional association matrix 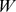 denotes the strength and direction of interaction from the channel *i* to the channel *j*.

### Graph Analysis

Graph analysis provides a relatively simple way to quantitatively analyze complex networks [29], which allows for a set of measures to quantify the characteristics of networks. Graph is a model to describe the system by defining a set of nodes and edges that represent the elements of system and their interactions. In the present study, the nodes represent a group of neurons around the recording sites and the edges represent the causal interactions among the nodes. The weight of each edge is the element in the association matrix denoting the interaction strength of the two nodes it connected.

The effective connectivity between neural groups in the hippocampal slices was investigated. Several measures were employed to characterize the neural network topology of hippocampal slices, which are defined as follows.

Degree: the degree of a node in a directed network consists of the in-degree and the out-degree. The in-degree of node *i* (*id_i_*) is defined as the sum of the weights of edges pointing towards 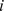 (i.e., inward edges). The out-degree of node *i* (*od_i_*) is accordingly defined as the sum of the weights of edges originating from 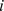 (i.e., outward edges). Formally,
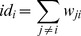
(8)and
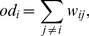
(9)where 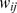 is the weight of edge 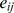 in the network. The total weighted degree of a node is the sum of its in- and out-degree. The connection strength of the network was computed as the sum of degrees of all nodes in the network. In the hippocampal neural network, a node with large in-degree and small out-degree indicates that it is an information flow-in site, and a node with small in-degree and large out-degree indicates that it is an information flow-out site. The distribution of the nodes with different in-degrees and out-degrees might indicate the information flow in the network.

In addition to the weighted degree, another degree definition, combinatorial degree [30] is used to define the clustering coefficient. The combinatorial degree of node 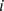 is the number of edges connected it (including inward and outward edges).

Clustering coefficient: the clustering coefficient of node 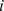 (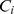) in a weighted directed network is defined as follows [31]:
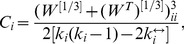
(10)where 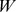 is the 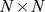 association matrix, 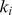 is the combinatorial degree of node 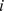, 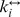 is the number of the bidirectional connections between the node 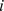 and the other nodes. The average clustering coefficient (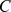) is defined as the average of 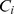 across all nodes in the network:
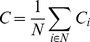
(11)


The clustering coefficient 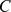 quantifies the local interconnectivity of the network. The random networks have low average clustering coefficients, whereas the complex networks have high average clustering coefficients that indicate the high local efficiency of the information transfer.

Path length: the path length between nodes 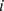 and 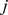 is defined as sum of the edge lengths along the path, where each edge's length is obtained by computing the reciprocal of the edge weight 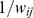. The shortest path length 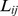 between nodes 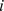 and 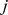 is defined as the length of the path with the shortest length between the two nodes. The characteristic path length (L) of the network is measured by the “harmonic mean” length between all of the node pairs as follows:
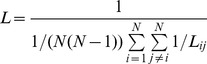
(12)The characteristic path length quantifies the ability of parallel information transfer and can reflect the efficiency of information transfer.

Small-world property: the small-world property was originally proposed by Watts and Strogatz [32]. The network that exhibits small-world property is one kind of the complex networks.

A small-world network has the similar characteristic path length but higher clustering coefficient compared to a random network, namely, the normalized characteristic path length 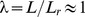 and the normalized clustering coefficient 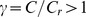, where 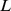 and 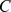 are the characteristic path length and the clustering coefficient of the network, the subscript 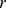 denotes the random network. Those two conditions can also be summarized into one quantitative measurement, the small-worldness, 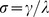, which is typically >1 for the network with a small-world organization [33]. The small-world organization is economical in the sense of providing high global and local efficiency of parallel information processing for low connection density [33].

Previous studies indicated that the brain network of each patient normally differed in both the number and the weighting of its edges [34]. Because the small-world property depended on the connection density of the networks, the small-worldness of each network should be compared at the same cost. Here the cost reflected the connection density of a network and was defined as the number of edges divided by the maximum possible number of edges [33], [35]. The small-world property of networks was inspected and compared at the same cost ranging from 0.05 to 0.3 by a step of 0.01 to insure an appropriate network density [11], [35].

To measure the small-worldness of the hippocampal neural network, 100 random directed weighted networks with the same number of nodes and edges were constructed [36], [37], which preserved the out-degree distribution of the original networks, and 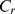 and 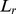 were defined as the average clustering coefficient and characteristic path length of the 100 random networks. Then, 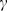, 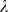 and 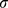 were calculated to evaluate the small-worldness of the hippocampal networks.

### Statistical Analysis

The analyses were made on the data collected from 8 slices. Only one slice was taken from each rat. The statistical analysis results are expressed as the means ± SEM. Statistical comparisons were made using Student's t-test, and *P*<0.05 was accepted as significant. The false discovery rate was used to correct the multiple comparisons [38].

## Results

### Mg^2+^-free-ACSF-induced Epileptiform Discharges

In our experiments, the application of Mg^2+^-free ACSF consistently induced synchronous epileptiform discharges across the hippocampal slices (Fig. 1B and C), which consisted of multi-unit activities (MUAs, >200 Hz) superimposed on local field potentials (LFPs, 1–200 Hz). The first epileptiform discharge appeared at 20.0±17.0 min after the onset of the Mg^2+^-free ACSF perfusion, and then the epileptiform discharges occurred at a frequency of 4.90±2.44 per minute (*n* = 8 slices). The epileptiform discharges recorded by the same electrode had similar amplitudes and waveforms (Fig. 1C and D) during the Mg^2+^-free ACSF perfusion. When the epileptiform discharges were steadily appeared (usually 30 min after the onset of Mg^2+^-free ACSF perfusion), 50 epileptiform discharges (each contained 800-ms recording data) were sampled from each electrode. To investigate the possible network alteration during the transition from normal to epileptiform discharges, each 800-ms data segment (76 windows with 50-ms-long window size and 10-ms-long sliding step) was divided into 3 stages: early, middle and late stage of epileptiform discharges, corresponding to 0–240 ms (window #1-#20), 280–520 ms (window #29–#48) and 560–800 ms (window #57–#76) of the 800-ms data segment, respectively (Fig. 1D).

### Information Flow in Hippocampal Network

**Figure 2:**
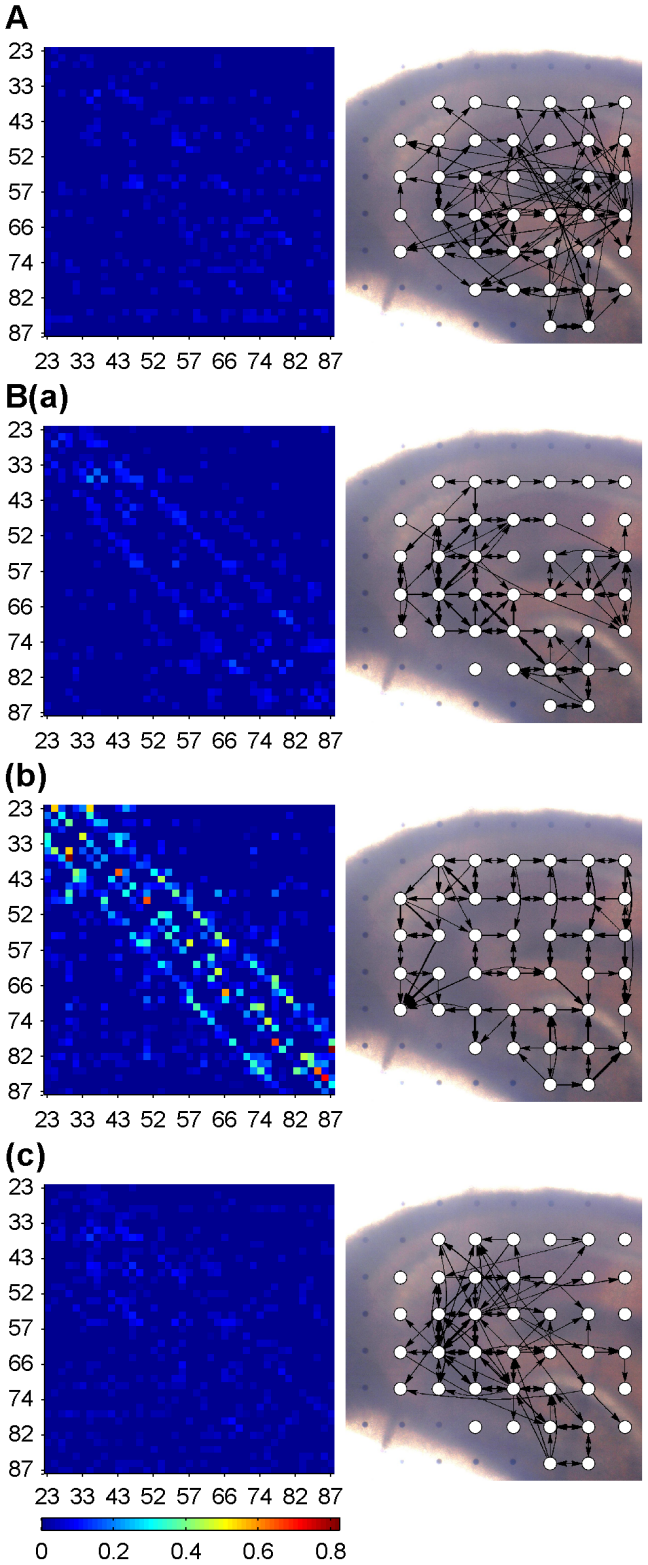
Association matrix (left) and connectivity graph (right) of the networks during control and epileptiform discharges. A: Association matrix and connectivity graph at window #1 of control state. B (a), (b), (c): Association matrices and connectivity graphs at windows #1, #35 and #76, which represent three stages of the epileptiform discharges. Coordinates of association matrix correspond to channel numbers (arranged in columns) shown in Fig. 1B. In the connectivity graphs, the direction and strength of interactions between any two nodes (white dots) are portrayed using arrows and their line widths. Only edges with the largest 100 weights in the corresponding association matrix are shown.

The interactions of neural activities in hippocampal slices were derived by PDC, using 50-ms-long windows of the data segments during control and epileptiform discharges. The association matrix and connectivity graph of one representative control network are shown in Fig. 2A. For clarity, only the 100 connections with large weights among all 41 nodes are shown. In control, the interactions among the neural groups in different recording sites appeared to be random and the network was dominated by long-distance connections. The association matrices of all 76 windows had similar random patterns. During the epileptiform discharges, the network organizations in different stages were distinct. The representative networks in the early stage (window #1, Fig. 2B(a)), middle stage (window #35, Fig. 2B(b)) and late stage of epileptiform discharges (window #76, Fig. 2B(c)) are shown in Fig. 2B. All of the association matrices of the three networks showed large weights in their diagonal directions (left of Fig. 2B). According to the arrangement of electrode numbers, the weights in the brightly-colored diagonals of the association matrices (left of Fig. 2B, especially B(b)) correspond to the edges that connecting horizontally or vertically adjacent nodes (right of Fig. 2B). Hence all of the three networks showed some regularity, i.e., many connections were formed in local areas. Most weights of the network in the middle stage of epileptiform discharges were much larger than those in the early and late stages. In the early and late stages of epileptiform discharges, there were no apparent synchronous discharges, whereas the networks in these two stages exhibited some regularity which was obviously different from the random organization of the network in control. During the epileptiform discharges, the nodes in the granule cell layer were connected to the other nodes with the edges mostly pointing to rather than departing from them (right of Fig. 2B). Similar observations were obtained from the other 7 slices. The network properties discussed here as an example would be further analyzed quantitatively and statistically in the following sections.

**Figure 3:**
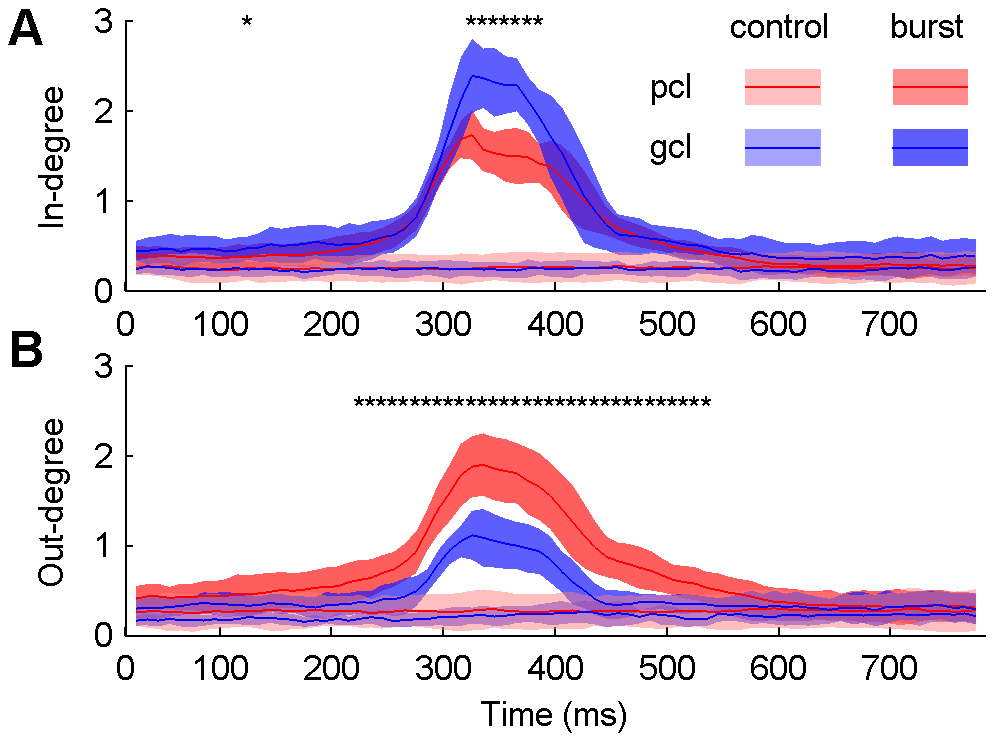
The in- and out-degree of nodes in hippocampal networks. Averaged in-degree (A) and out-degree (B) of the nodes in the pyramidal cell layer (pcl) and the granule cell layer (gcl) of the hippocampal slice in control (light shades and lines) and epileptiform discharges (dark shades and lines). Lines denote mean values and shades denote standard errors. Differences of the averaged in-degree/out-degree of nodes in the pcl and the gcl during epileptiform discharges are tested (*P<0.05, *n* = 8, paired t-test with FDR correction).

After removing the spurious links by the statistical significance test, the in- and out- degrees of all nodes in each network at each time window during control and epileptiform discharges were obtained. Statistical analysis results of 8 slices are shown in Fig. 3. The averaged in-degree and out-degree of the networks were steady at all time windows in control. There was no difference of the in-degree or out-degree between the nodes in the pyramidal cell layer (pcl) and the granule cell layer (gcl). However, during epileptiform discharges, both of the averaged in-degree and out-degree of the network were increased. The averaged in-degree of the nodes in the pcl was smaller than that in the gcl (Fig. 3A). Meanwhile, the averaged out-degree of nodes in the pcl was larger than that in the gcl (Fig. 3B). There was no significant difference between the averaged in-degree or out-degree in the pcl and the gcl in most of the time widows in the early and late stages of epileptiform discharges. The averaged out-degree in the gcl was smaller than that in the pcl in the whole middle stage of epileptiform discharges, whereas the difference in the in-degree of the pcl and the gcl was only significant in the center period of the middle stage of the epileptiform discharges (*P*<0.05, *n* = 8, paired t-test with FDR correction).

These results indicated that during epileptiform discharges, the connection strength of the hippocampal neural network was increased. The large out-degree, small in-degree of nodes in the pcl vs. large in-degree and small out-degree of nodes in the gcl indicated that there was net information flow from the pcl to the gcl, with the nodes in the pcl being sources and the nodes in the gcl being sinks. There was no apparent net information flow between the pcl and the gcl observed in networks in control, which was consistent with the property of random networks.

### Small-world Property

To inspect the small-world property of networks and its possible alteration during epileptiform discharges, the small-world property in each stage was inspected. The normalized clustering coefficient (γ), normalized path length (λ) and small-worldness (σ) of networks within each stage of epileptiform discharges were similar, and the averaged values in each stage were used to characterize the networks. Because the small-worldness was related with the network connection density (cost), different costs were applied to access the small-worldness of the networks.

**Figure 4:**
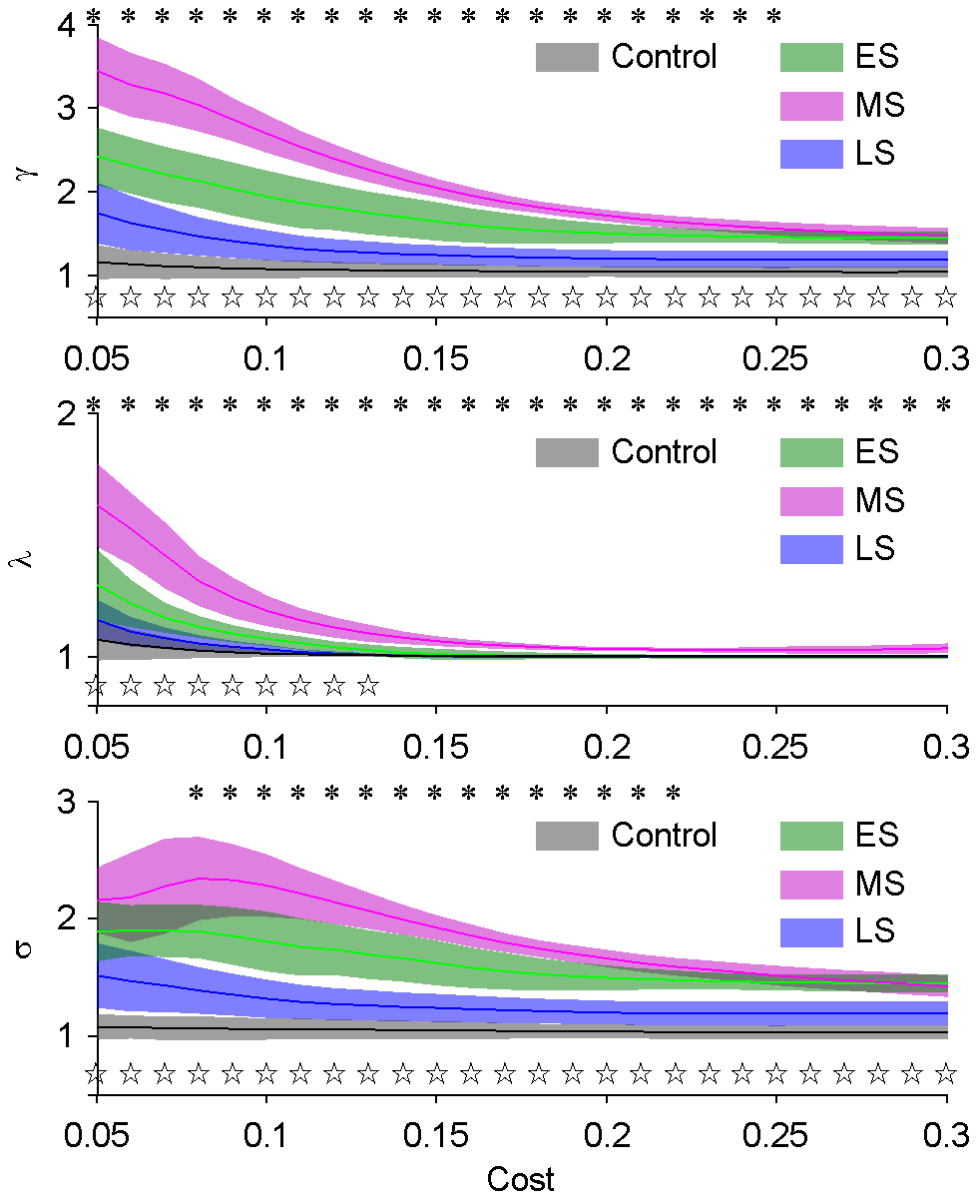
Small-world property of hippocampal networks at different time stages. Normalized clustering coefficient (γ), normalized characteristic path length (λ) and small-worldness (σ) (top to bottom, respectively) of control and 3 stages of epileptiform discharges (early, middle and late stages of epileptiform discharges, abbreviated as ES, MS and LS) as a function of the cost. Lines denote the mean values and shades denote the standard errors. Difference of σ, γ and λ between early stage and middle stage are tested (*P<0.05, *n* = 8, paired t-test with FDR correction). Difference of σ, γ and λ between early stage and late stage are also tested (*P<0.05, *n* = 8, paired t-test with FDR correction).

The networks in all three stages of epileptiform discharges showed the small-world property, whereas the networks in control did not. The results are shown in Fig. 4. The network in control had the similar average clustering coefficient and characteristic path length as random networks. However, the networks in all three stages of epileptiform discharges exhibited the small-world property. The normalized clustering coefficient (γ) and normalized path length (λ) were larger than 1 during epileptiform discharges, which indicated that the networks had more local connections as well as less long-range connections than random networks. The results demonstrated that the network shifted from a random organization towards a more regular (lattice-like) organization during the transition from normal to epileptiform discharges. Additionally, the network organizations in different stages of the epileptiform discharges were discrepant. The relation 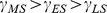 and 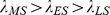 (MS: middle stage, ES: early stage and LS: late stage of epileptiform discharges) indicated that during the transition process from normal to epileptiform discharges, the network underwent increase in local connections and decrease in long-range connections, and then, in the late stage of epileptiform discharges, the local connections decreased and the long-range connections increased, and the network shifted towards a more random pattern than the network in the early stage of epileptiform discharges.

### Time Courses of Epileptiform Discharges and Network Alteration

The recurrent epileptiform discharges in hippocampal slices were induced by Mg^2+^-free ACSF, meanwhile networks topology changed during this process. The preceding results showed that the networks in the early and late stages of epileptiform discharges exhibited some regularity and had the small-world network property, whereas the networks in control were randomly organized and did not exhibit the small-world property. On the other side, in the early and late stages there were no apparent epileptiform discharges and the signals were similar to those in control. These results suggested that there might be inconsistencies between the time courses of the epileptiform discharges and the network alteration.

To compare the time courses of the epileptiform discharges and the network alteration, longer data segments (7 s, 2 s pre- and 5 s post- the detected time point of epileptiform discharges) of the epileptiform discharges were analyzed in the same way as the previous procedure. The ensemble mean of the 50 epileptiform discharges in each channel was calculated at each time point. The averaged waveform was then smoothed (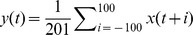, where 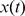 and 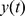 are the waveforms before and after smoothing). The start and end time were determined using a threshold of ±4 times the standard deviation of the baseline of the smoothed waveform. The start and end time were the first and last time points when the smoothed waveform crossed the threshold. Compared among all channels, the earliest and latest time were identified as the start and end time of epileptiform discharges in the whole network, respectively.

**Figure 5:**
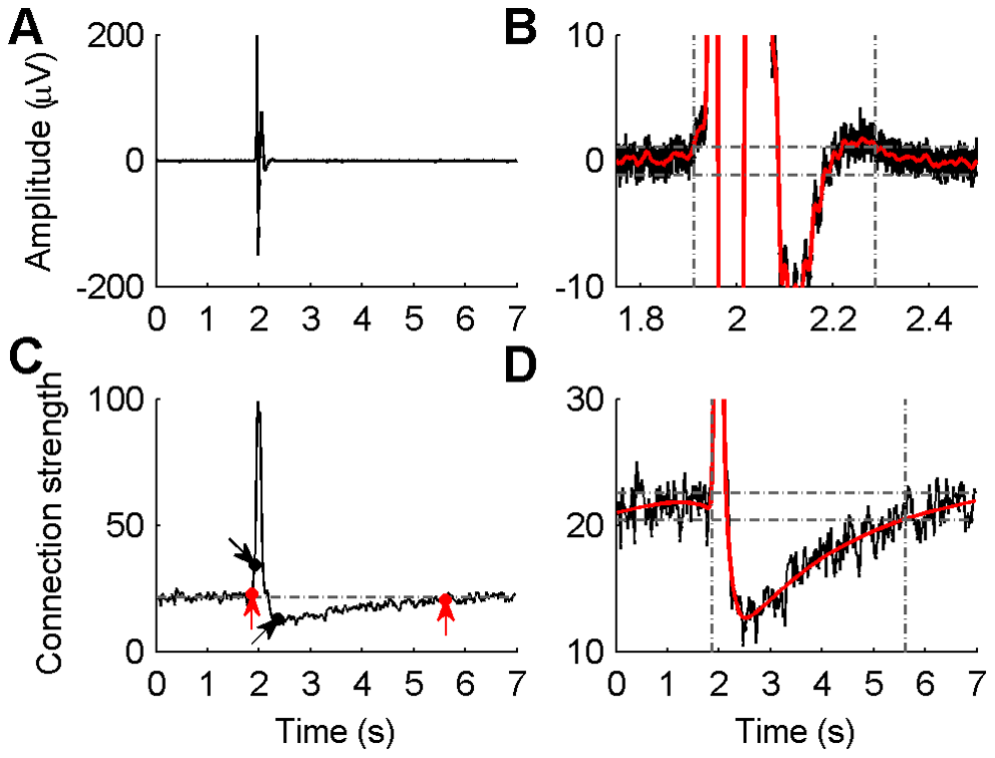
Time courses of epileptiform discharges and network alteration. A: Typical epileptiform discharges averaged across 50 epileptiform discharges recorded by one channel. B: Detection of the start and end time of the averaged signals. After detecting the start and end time in each channel, the earliest start time and the latest end time across all channels were identified as the start and end time of epileptiform discharges in the whole network. C: Network connection strength during epileptiform discharges. Two black dots with arrows indicate the start and end time of epileptiform discharges, and two red dots with arrows indicate the start and end time of the network alteration. D: Detection of the start and end time of the network connectivity alteration. In (B) and (D), red curves are smoothed signals, horizontal dashed lines are thresholds and vertical lines indicate the start and end time detected.

The network connection strength was computed in all windows during the epileptiform discharges. The results were smoothed using the Bayesian adaptive regression splines (BARS [39]) as shown in Fig. 5D. A threshold of ±4 times the standard deviation of the baseline of the smoothed connection strength was set. The first and last windows that the smoothed curve exceeded the threshold were detected and their corresponding time points were regarded as the start and end time of the network alteration.

The start and end time of the epileptiform discharges and the alteration of network connection strength were compared. The result demonstrated that the alteration of network connection strength started earlier than the epileptiform discharges appeared, and such network alteration was kept unrecovered even when the epileptiform discharges ceased. The leading and delay time of the network alteration as compared to the epileptiform discharges change were 0.19±0.17 s and 3.61±0.92 s, respectively (*n* = 8 slices). The results illustrated that the effective connectivity of the hippocampal network started to change before the appearance of the epileptiform discharges. The network reorganization and the enhancement of the network connection strength might underlie the generation of epileptiform discharges in the hippocampal network. When epileptiform discharges ceased, the network connection strength did not recover immediately, but underwent a slow recovery process that lasted for several seconds. During the time episode between each two epileptiform discharges, the network had stronger connection strength as compared to that in control (data not shown).

## Discussion

In our present study, the graph theory was applied to investigate the effective connectivity of rat hippocampal neural network and its alteration during the transition from normal to the epileptiform discharges induced by Mg^2+^-free ACSF. The results showed that neural groups in different layers and regions of hippocampal slices exhibited distinct network properties, which might reflect the different functional roles in each region. The networks showed the small-world organizations and were more regular during epileptiform discharges, whereas the networks in control were random networks. These results might be helpful for understanding the characteristics of hippocampal neural network and its alterations related to epilepsy. On the other hand, the network analysis results showed that the network property changes occurred before the detectable epileptiform discharges appeared, and the network properties did not recover immediately to the control when the epileptiform discharges ceased. So, the network analysis might provide a meaningful tool to evaluate the neural network and its alteration during the transition from normal to epileptiform discharges.

### Model Order Determination

**Figure 6:**
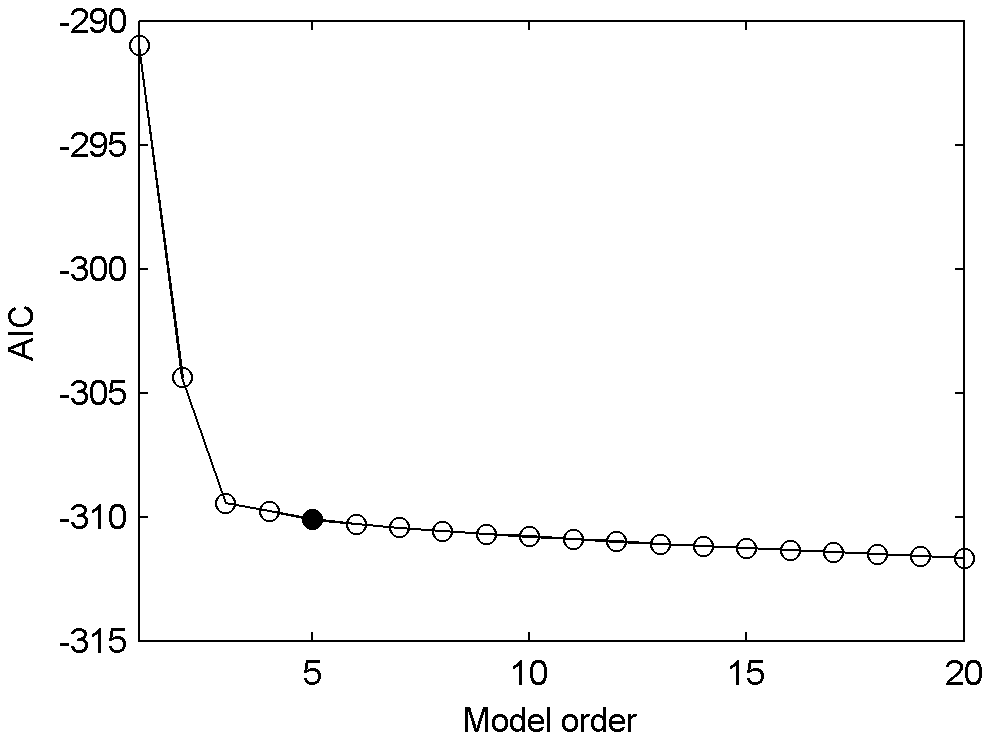
Model order determination. The AIC value as a function of the model order, computed in a representative 50-ms window of epileptiform discharges. The shape of the curve is similar for all windows during control and epileptiform discharges.

One of the problems in MVAR modeling is to choose the model order, which could be determined by the AIC. Fig. 6 shows the AIC value as a function of the model order for our representative multivariate signals. The AIC value decreases monotonically with increasing order, which is consistent with MVAR modelings applied on EEG time series [23]. The decreasing rate of AIC value was very slow when the model order was larger than 4, so we applied a 5th-order MVAR to fit our data. The model orders of 3–10 were additionally applied to test the fitting effect, and the results demonstrated good tolerance of the process to different model orders (data not shown).

### Weighted and Directed Graphs

Unweighted links in a network indicate the presence or absence of connections between nodes, which are usually obtained by applying a threshold to the weighted network. The weight is set to 1 when it is larger than the threshold, otherwise it is set to 0. To date, most network studies using graph theory construct unweighted graphs [29], [40]. Recently the weighed network has been used to explore the neural network characteristics, such as the seizure onset zone of epileptic patients [7], [8] and the network topology of human brain in physical and pathological conditions [11], [41].

Graphs can also be formed with directed or undirected edges. The directed edges contain the information about the causal relations between nodes, whereas an undirected graph can only tell the existence of connections [40]. Thus, the directed graph might be more valid when modeling causal interactions among neural groups. The human neuroimaging data are difficult to assign directionality to the networks [40], which might be the result of the low time resolution of the signals. However, the electrical signals recorded by multiple microelectrodes are feasible for constructing the directed graphs. The weighted and directed network analyses were effective in identifying the seizure onset zone in the cortices of epilepsy patients [7], [8]. In the present study, we investigated the characteristics of hippocampal neural network using the weighted and directed graph, which made the network analysis more realistic.

### Information Flow

Studies on the information flow by inspecting the effective connectivity had been carried out within the hippocampus as well as between the hippocampus and the other cortex regions. Korzeniewska et al. investigated the information flow between the hippocampus and the other related structures during various types of rat's behaviors. The result demonstrated that during different behavior tasks, the information flow among the different brain regions of rat has different strengths or directions [42]. Baccala et al. applied PDC analysis on EEG data collected from rats during the slow-wave sleep which had spindle episodes containing a burst of oscillatory brain activities [43]. Their result illustrated that the direction of the information flow between two brain regions changed before/after and during spindle episodes [43]. These studies indicated that under different physical conditions, behavioral tasks, etc., the information flow in neural network would change its connection status. In our study, the net information flow from the pcl to the gcl formed during the epileptiform discharges but not in control. This might be the result of increased excitatory of CA3 neurons, which influenced the granule cells due to the collaterals of CA3 pyramidal cells' back-projection to the DG during epileptiform discharges. However, in control, the back-projection from CA3 to dentate granule cells was normally suppressed by the GABAergic inhibition, so CA3 did not have a robust excitatory influence on the granule cells [44]. Zhang et al. investigated the excitatory synaptic input to the granule cells from CA3 regions in pilocarpine-treated rats, and the result demonstrated that the stimulation on the proximal CA3 pcl were more likely to evoke the excitatory postsynaptic currents in the granule cells of the epileptic rats as compared with control rats [45]. Furthermore, the synaptic strengths of recurrent excitatory inputs to the granule cells from CA3 pyramidal cells were increased in epileptic rats [45]. These evidences might give an explanation for the increased information flow from the pcl to the gcl in our research.

### Small-World Property

The results of graph analysis showed that the neural networks during control and epileptiform discharges exhibited different small-world property. The random network organization was observed in control, whereas the small-world architecture appeared during epileptiform discharges. During epileptiform discharges, the hippocampal networks showed significant increases of normalized clustering coefficient (γ), which indicated that the network formed more local connections as compared to the control. It denoted an alteration from a random architecture in the normal network to an organized architecture in the pathological networks. Some studies on the neural network topology of human brain using EEG data (including intracranial or intracerebral EEG) demonstrated the increased small-world organization or more regular network configuration in epileptic brains as compared with normal or less pathological controls [46]–[49]. However, some other studies showed the disrupted or decreased small-world organization in epileptic brains [9], [11] or epileptiform network of cultured hippocampal neurons [50]. Our study on hippocampal network in Mg^2+^-free epilepsy model supported the former result. Moreover, scale-free organization was observed in developing hippocampal networks [22]. These discrepancies in the network topology and its alteration during epileptiform discharges might be due to the different experimental preparations or epilepsy phenotypes, etc. Additionally, it reflects the demands of further understanding on the organizational mechanism of the complex neural network.

### Epileptiform Discharges and Alteration of Network Organization

The study on the time courses of the epileptiform discharges and the network alteration indicated that the enhancement of the network connection strength might underlie the epileptiform discharges in rat hippocampal slices. According to the exploration results of small-world property of the network, the enhancement of connection strength might be the result of the rapid increase of connections among adjacent neural groups. This might involve the increased neuronal excitability due to the activation of N-methyl-D-aspartate (NMDA) receptors during the perfusion of Mg^2+^-free ACSF. During the epileptiform discharges after the perfusion of Mg^2+^-free ACSF, the glutamate released from the presynaptic neuronal terminals could generate larger excitatory postsynaptic potentials by both NMDA and non-NMDA receptor-gated ion channels than by non-NMDA receptor-gated ion channels only [20]. It indicated that larger influence of presynaptic neurons would be exerted on the postsynaptic neurons and formed a comprehensive effect of increased connection strength in the network during the perfusion of Mg^2+^-free ACSF. After a period of enhancement, the network connection strength decreased to a level lower than that in the early stage of epileptiform discharges and then recovered to the baseline in several seconds. The shape of the recovery procedure and its time course were similar to the after-hyperpolarization (AHP) of the interictal spike recorded intracellularly in the hippocampal pyramidal cell [51], [52]. It is possible that the recovery procedure reflected the AHP of the interictal spike. This conjecture might be tested by blocking the AHP and observing the recovery procedure, which would be one of the research subjects in pharmacological studies in the future. The network analysis reflected the network alteration that was not revealed by the signal waveform. This manifested the advantages of the network analysis in exploring the network characteristics and its potential applications in seizure prediction, seizure foci localization, and so on.

## Conclusions

In this study, the network characteristics of hippocampal slices and their alterations during the transition from normal to epileptiform discharges were investigated. The effective connectivity of hippocampal neural network was formed by the PDC, and the network characteristics were analyzed based on the graph theory. The results showed that during epileptiform discharges, the nodes in the pcl had large out-degree and small in-degree, whereas the nodes in the gcl had large in-degree and small out-degree. This indicated that there was net information flow from the pcl to the gcl during epileptiform discharges, which did not exist in control. The network exhibited random connections in control, whereas it showed a small-world organization during epileptiform discharges, and the increased local connections contributed this transition. Additionally, we found that the network alteration occurred prior to the start of the epileptiform discharges and posterior to the end of the epileptiform discharges. These results revealed several important alterations of the network characteristics during the transition from normal to epileptiform discharges. This might provide new insights into the hippocampal neural network and epilepsy mechanisms. The study manifested the advantage of the network analysis method in analyzing the network characteristics and might help to improve the prediction of seizures.
